# Efficient evaluation of a gene containment system for poplar through early flowering induction

**DOI:** 10.1007/s00299-020-02515-1

**Published:** 2020-02-12

**Authors:** M. Valentina Briones, Hans Hoenicka, Luis A. Cañas, José Pío Beltrán, Dieter Hanelt, Sandra Sharry, Matthias Fladung

**Affiliations:** 1 Facultad de Ciencias Agrarias y Forestales, Universidad Nacional de La Plata, B1900 La Plata, Argentina; 2 Thünen Institute of Forest Genetics, 22927 Grosshansdorf, Germany; 3 Instituto de Biología Molecular y Celular de Plantas (CSIC-UPV), 46022 Valencia, Spain; 4 Institut für Pflanzenwissenschaften und Mikrobiologie, Universität Hamburg, 22609 Hamburg, Germany; 5 Laboratorio de Investigaciones de la Madera (LIMAD), Facultad de Ciencias Agrarias y Forestales, Universidad Nacional de La Plata, B1900 La Plata, Argentina; 6 CIT-Viedma, Universidad Nacional de Río Negro, R8500 Viedma, Argentina; 7 Consejo Nacional de Investigaciones Científicas y Técnicas (CONICET), B1900 La Plata, Argentina; 8 Comisión de Investigaciones Científicas de la Provincia de Buenos Aires (CIC), B1900 La Plata, Argentina

**Keywords:** Gene containment, Early flowering, Genetic transformation, *Populus*, *PsEND1*, Biosafety

## Abstract

**Key message:**

The early flowering system HSP::*AtFT* allowed a fast evaluation of a gene containment system based on the construct PsEND1::*barnase–barstar* for poplar*.* Transgenic lines showed disturbed pollen development and sterility.

**Abstract:**

Vertical gene transfer through pollen flow from transgenic or non-native plant species into their crossable natural relatives is a major concern. Gene containment approaches have been proposed to reduce or even avoid gene flow among tree species. However, evaluation of genetic containment strategies for trees is very difficult due to the long-generation times. Early flowering induction would allow faster evaluation of genetic containment in this case. Although no reliable methods were available for the induction of fertile flowers in poplar, recently, a new early flowering approach was developed. In this study, early flowering poplar lines containing the gene construct PsEND1::*barnase–barstar* were obtained. The PsEND1 promoter was chosen due to its early expression pattern, its versality and efficiency for generation of male-sterile plants fused to the *barnase* gene. RT-PCRs confirmed *barnase* gene activity in flowers, and pollen development was disturbed, leading to sterile flowers. The system developed in this study represents a valuable tool for gene containment studies in forest tree species.

**Electronic supplementary material:**

The online version of this article (10.1007/s00299-020-02515-1) contains supplementary material, which is available to authorized users.

## Introduction

The demand of wood from forests, orchards, and plantations is estimated to increase threefold by 2050 (WWF 2015), mostly as a result of global population growth, which will greatly affect forest biodiversity and wood-based products (Fritsche et al. 2018). Technological advances in silviculture, land management, and generation of biotech-based tools will lead to an improvement in productivity and sustainability of natural and planted forests (Al-Ahmad 2018). Unfortunately, forest trees are long-living and characterized by extended vegetative growth phases, thus impeding rapid progress in forest tree breeding (Brunner et al. 2017). Genetic engineering and gene editing (GE) are valuable tools for bypassing the long-generation times in trees, allowing traits to be added or modified without significant background changes to commercially valuable clones (Klocko et al. 2018). Sterile trees are appealing to consumers who are against GE trees, and influence a number of obstacles faced when attempting to increase productivity from planted forests (Fritsche et al. 2018). The development of biotechnological tools to engineer sterility in forest trees, the so-called “gene containment” methods, can mitigate the gene flow from genetically engineered commercial plantings to wild relatives, as well as the damage caused by pollen and seed escapes from planted forests of non-native invasive woody species. The current availability of genomics and transcriptomics resources as well as new breeding technologies, including gene editing, has led to a gain of knowledge in the genetic control of reproductive processes of both hardwood and softwood tree species (Fritsche et al. 2018).

Different biotechnological methods based on the ablation of reproductive structures or the inactivation or suppression of floral organ identity genes have been reported (reviewed in Fritsche et al. 2018). Engineered male sterility has been achieved in both Angiosperm and Gymnosperm trees by expression of the *barnase* gene from *Bacillus amyloliquefaciens* (Paddon and Hartley 1986) under the control of different reproductive tissue-specific promoters. Furthermore, *barstar*-carrying transgenic lines (encoding a Barnase inhibitor) have been shown to restore male fertility when crossed with Barnase-induced sterile plants (Hartley 1988; Mariani et al. 1992; Roque et al. 2019). The use of *barnase* in combination with the *barstar* gene protects plants from improper ribonuclease expression (Gardner et al. 2009). Cell ablation was reported in Angiosperm trees in the inflorescences of *Betula pendula* using the construct BpFULL1::*barnase* (Lännenpää et al. 2005)*,* in *Eucalyptus occidentalis* trees male flowers using the construct PrMC2::*barnase-H102E* (Zhang et al. 2010), and in *Populus tremula* × *P. tremuloides* trees in the tapetum using the TA29::*barnase* construct (Elorriaga et al. 2014). Klocko et al. (2018) produced male-sterile *P. tremula* × *P. tremuloides* trees by RNAi gene silencing of *LEAFY* (*LFY*)*/AGAMOUS* (*AG*). The recently characterized *SEPALLATA3* gene promoter from London plane (*Platanus acerifolia*) in combination with the *barnase* gene has been proposed for sterility induction in plants (Lu et al. 2019). In Gymnosperm trees, male-sterile *Pinus rigida* × *P. taeda* trees were obtained by cell ablation of male cones using the construct *PrMC2–barnase–H102E* (Zhang et al. 2010)*.* Site-directed mutagenesis of flowering-time genes via CRISPR-Cas9 has been demonstrated in both male and female poplar genotypes (Elorriaga et al. 2018).

The expression pattern of the pea anther-specific gene *PsEND1* (*Pisum sativum ENDOTHECIUM 1*) has been assessed in both model plants (*Arabidopsis thaliana* and *Nicotiana tabacum*) and crops (tomato, oilseed rape, tobacco, rice, and wheat) using genetic constructs carrying the *PsEND1* promoter driving the *uidA* reporter gene (Gómez et al. 2004). Cell ablation in the anther tissues has been shown using this promoter driving the *barnase* gene in several plant species, leading to prevention of mature pollen grains production in all cases (Roque et al. 2007, 2019; Beltrán et al. 2007; Pistón et al. 2008; García-Sogo et al. 2010, 2012; Medina et al. 2013; Rojas-Gracia et al. 2017). Early anther cell ablation in the *PsEND1*::*barnase–barstar* plants result in additional effects with potential biotechnological applications including production of hybrid seed, transgene bioconfinement in genetic modified crops, redirection of resources to increase vegetative growth (Beltrán et al. 2007), elimination of pollen allergens in transgenic ornamental plants such as *Kalanchoe* (García-Sogo et al. 2010) or *Pelargonium* (García-Sogo et al. 2012), and production of seedless parthenocarpic tomatoes (Medina et al. 2013; Rojas-Gracia et al. 2017, 2019). The *PsEND1::barnase–barstar* construct has not been assayed so far in woody plant species.

The reproductive phase in forest trees is only reached after prolonged juvenile growth ranging from many years to even decades (Weigel and Nilsson 1995; Hoenicka and Fladung 2006), resulting in extremely long-generation times to conduct gene containment studies. Such studies must be performed in the frame of field trials, and results can be expected only many years or even decades later (Elorriaga et al. 2014; Klocko et al. 2016, 2018). The achievement of efficient early flowering systems would allow faster studies on genetic containment with trees species. However, first, biosafety studies following this strategy with poplar showed the importance of improving early flowering systems (Meilan et al. 2001; Hoenicka et al. 2006, 2012).

Different early flowering strategies based on the genetic transfer of a flowering-time gene driven by a gene promoter, e.g., heat-shock (*HSP*) and 35S promoter, have been proposed for poplar. However, no fertile flowers were reported with those approaches (Weigel and Nilsson, 1995; Rottmann et al. 2000; Böhlenius et al. 2006; Hsu et al. 2006; Tränkner et al. 2010; Zhang et al. 2010; Xiaoming et al. 2011; Shen et al. 2012; Xiaoming and Huanling 2014; Parmentier-Line and Coleman 2016). Pollen grains were reported in some flowers of 35S::*AtLFY*-poplars growing in a greenhouse (Hoenicka et al. 2006). However, pollen development was disturbed, flowers often lacked pollen grains, and flower fertility could not be confirmed with crossings. The first reliable system for induction of fertile flowers in poplar was developed by our team (Hoenicka et al. 2016). This system, based on the *HSP::AtFT* gene construct (Hoenicka et al. 2016), requires a heat-shock phase for *AtFT* expression induction followed by a cold-treatment phase for the activation of pollen development. Pollen grains can be obtained under both long- and short-day conditions in male and female plant flowers, indicating that the influence of the photoperiod on flower fertility was not equivalent. When transgenic female early flowering poplar were crossed and evaluated after 8 weeks of inductive treatments, all mature flowers obtained were fertile. The expression of poplar genes homologous to pollen development genes from *Arabidopsis thaliana* was affected by cold temperatures. A role for *PtTDF1*, *PtBAM1*, *PtSERK1/2*, and *PtMS1* on anther and pollen development in poplar flowers has been suggested based on homology and gene expression patterns. The non-reproductive phase of about 7–10 years was considerably shortened to 6–10 months using this system, which additionally allows season-independent production of fertile flowers (Hoenicka et al. 2016).

Here, we report the production and characterization of genetically modified poplar (*P. tremula* L.) lines containing two gene constructs (HSP::*AtFT* and PsEND1::*barnase–barstar*) with evaluation of early flowering and male sterility. We could show that the PsEND1 promoter efficiently transcribes the *barnase* gene in poplar flowers leading to male-sterile plants. This system based on early flowering transgenic lines is an efficient and reliable tool for gene containment research in poplar.

## Materials and methods

### Generation of the pBI101-PsEND1::*barnase–barstar* construct

The pBI101–PsEND1::*barnase–barstar* construct was generated in a plasmid derived from pBI101 harbouring, from the right to the left border, the *nptII* marker gene under the control of the *nos* promoter and the *nos* terminator, and the *barnase–barstar* gene under the control of the *PsEND1* promoter and the *nos* terminator (Roque et al. 2007). The promoter region (−2736/−6) of the *PsEND1* gene (GenBank Accession: AY324651) was previously cloned into the binary vector pBI101. Primers Ribo1 (5′-TAGGATCCCGACCATGGCACAGGTTATC-3′) and Inhi2 (5′-GCGAGCTCTTAAGAAAGTTGATGGTGATG-3′) were designed based on the published sequence of *barnase* and *barstar* genes (Hartley 1988) to amplify the *barnase–barstar* fragment (800 bp) and to introduce *Bam*HI and *Sac*I restriction sites. Between the *barnase* and *barstar* genes, there is a non-codifying sequence. Barnase is a very active ribonuclease, and thus, even a low level of expression from aberrant promoter sequences or run-off expression from neighbouring genes during manipulation in *E. coli* or *Agrobacterium* would have prevented the survival of the bacteria. Therefore, the *barstar* gene which encodes an inhibitor of Barnase is included in the construct. The PCR resulting fragment was cloned into the pGEM-T Easy (Promega) and later released with the *Bam*HI and *Sac*I enzymes. The *Bam*HI*–Sac*I fragment was cloned into the pBI101-*PsEND1* generating the pBI101-PsEND1::*barnase–barstar* gene construct. The nos::*nptII* plant selectable marker gene, which confers resistance to kanamycin in transgenic plants, was also introduced in the T-DNA fragment.

### Plant material, vectors, and genetic transformation

Transformation vectors pk2GW7:HSP::*AtFT* and pBI101:END1::*barnase–barstar* were introduced into the *Agrobacterium* strains GV2260 and EHA105, respectively. In vitro cultures of two male clones (*P. tremula* L. × *P. tremuloides* Michx. clone T89 and *P. tremula* L., clone W52) were used for the generation of the transgenic lines. The plants were grown on solid McCown Woody Plant Medium (WPM, Duchefa M0220) (Lloyd and McCown 1980) containing 2% sucrose and 0.6% Agar (Agar, Serva, 11396). Genetic transformations were carried out employing the *Agrobacterium*-mediated approach (Fladung et al. 1997) using simultaneously two *Agrobacterium tumefaciens* (Smith and Townsend 1907) strains, one containing pBI101:END1::*barnase–barstar* and the other one pk2GW7:HSP::*AtFT*. For regeneration of transgenic plants, WPM medium was supplemented with 0.01% Pluronics F-68 (Sigma P-7061, Steinhein, Germany), thidiazuron (0.01 μM), and antibiotics cefotaxime (500 mg L^−1^) for Agrobacteria elimination and kanamycin (50 mg L^−1^) for selection of transgenic shoots. Plants were transferred to growth chambers (Weiss Technik, Reiskirchen, Germany) under the following culture conditions: light period 16/8 h (day/night), light irradiance 300 µmol m^−2^ s^−1^(lamps Phillips TLM 140 W/33RS, Amsterdam, The Netherlands), relative humidity 70%, and temperature 22/19 °C. After a culture period of 6–18 months in growth chambers, transgenic plants were transferred to a standard S1 greenhouse under natural daylight conditions. Studies were carried out at the Institute of Forest Genetics in Großhansdorf, Germany (Latitude: 53° 39′ 42.5952″ N, Longitude: 10° 15′ 12.7764″ E).

### Extraction of DNA and molecular analysis

Genomic DNA from male transgenic double and single lines and WT lines was extracted from in vitro grown leaves and buds. DNA extraction was followed by a standard protocol from Dumolin et al. (1995). DNA was quantified using spectrophotometric OD260 measurements with a Nanodrop 1000 (Thermo Scientific, Wilmington, DE, USA). Polymerase chain reaction (PCR) analyses were carried out to detect the transgenes using specific primers (Table S1 available as Supplementary Data) and annealing temperatures between 55 and 60 °C, as previously described (Hoenicka et al. 2012, 2014). Southern blot analyses were carried out with 20 µg genomic DNA digested with the restriction enzyme *Sac*I (Fermentas, Waltham, MA, USA), according to the supplier’s instructions. DNA electrophoresis and transfer of DNA to Biodyne A membranes (Pall Europe Limited, Portsmouth, UK) were performed as described elsewhere (Fladung et al. 1996, 1997). Southern blot prehybridizations and hybridizations with the non-radioactive DIG (digoxigenin) system were performed using a DIG-dUTP PCR-labeled probe as described earlier (Fladung and Ahuja 1995; Fladung et al. 1996). DIG probes were prepared with a PCR amplification Kit (PCR DIG Probe Synthesis Kit, Roche) using the different plasmids with the respective primer pairs (Table S1 available as Supplementary Data). Probe hybridization and chemiluminescent reaction were performed according to Roche instructions with some modifications (Fladung and Ahuja 1995). The gels were stained with Roti-Safe (Roth, Karlsruhe, Germany) shortly before blotting to confirm similar loaded DNA amounts and uniform restriction patterns.

### Induction of fertile flowers in transgenic poplar

Five-to-six-month-old greenhouse plants from a single transgenic line (T-193-2: fertility control), containing the HSP::*AtFT* gene construct, and six double transgenic lines containing both gene constructs HSP::*AtFT* and PsEND1::*barnase–barstar*, were subjected to treatments aiming at the promotion of fertile flowers. Flowering induction was carried out in growth chambers (Weiss Technik BioClim, Reiskirchen, Germany) as described before (Hoenicka et al. 2016). Plants were transferred at the end of the winter time from the greenhouse into the growth chambers. The induction of early flowering was carried out using two culture phases:

- Phase 1 (flower induction): plants were maintained under growth promoting conditions (day/night: 22/16 °C, 10/14 h) and heat treatments (40 °C, 90 min, 3–5 weeks) were applied daily until initiation of flower development.
- Phase 2 (fertility induction): plants were kept under cold conditions (day/night: 10/6 °C, 10/14 h) until full flower development, and no heat treatments were applied to plants.

### RNA extraction and reverse transcription

Selected flowers from five double and one single (early flowering control) transgenic lines were frozen in liquid nitrogen and stored at − 80 °C until RNA extraction. Around 70 mg of liquid nitrogen frozen tissue was ground in Eppendorf tubes using metal balls and a Retsch mill (Retsch MM300, Haan, Germany). Total RNA was isolated (Chang et al. 1993) and purified with the RNeasy MinElute Cleanup Kit (Qiagen, Hilden, Germany). RNA was spectrophotometric quantified with a Nanodrop 1000 (Thermo Scientific, Wilmington, DE, USA). RNA quality was assessed by OD260/OD280 and OD260/OD230 ratios (both were maintained between 1.8 and 2.1) and with the Agilent Bioanalyzer (Agilent Technologies Inc., Palo Alto, CA, USA). Samples with RIN values higher than 7 were selected. Contaminating DNA was removed from RNA samples using the Ambion turbo DNA-free (Ambion, Austin, TX, USA) according to the manufacturer’s protocol. The cDNA was synthesized with 2.31 µg RNA using the SuperScript VILO cDNA Synthesis Kit (Invitrogen, Carlsbad, CA, USA) according to the manufacturer’s instructions.

### Gene expression analysis of transgenic plants

Expression of *barnase* gene was studied using RT-PCRs. *UBQ7* (Accession: Potri.005G198700.1) was used as a reference gene. Specific primers (Table S1 available as Supplementary Data) were designed for both genes using Primer3Plus software (Rozen and Skaletsky 2000) with melting temperatures around 60 °C. PCR reactions were conducted in a 20 μl volume containing 300 nM of each primer, 2 µl of cDNA sample (~ 3.5 ng of input RNA), and Maxima Hot Start Taq DNA Polymerase (Fermentas, St. Leon-Rot, Germany). RT-PCRs were performed using the following parameters: 10 min at 95 °C and 40 cycles of 95 °C for 30 s, 60 °C for 1 min, and 72 °C for 1 min.

### Evaluation of pollen viability

Microspore viability was estimated by staining with fluorescein diacetate 0.01% (Wildholm 1972). Samples were incubated with the dye solution at RT for 15 min. The fluorescence was observed under an optical fluorescence microscope BH-2 (Olympus, Tokyo, Japan) microscope with a fluorescence equipment BH2-RFL (Olympus, Tokyo, Japan) using a mix of white (OLYMPUS 12V-100W HAL-L) and blue (490 nm, Osram HBO 100 W/2) light.

### Microscopic studies of anthers

Anthers from a single transgenic line (T-193-2: fertility control) and double transgenic lines, were studied microscopically. Samples were fixed (4% paraformaldehyde + 1% GA in 50 mM MSB buffer pH 6.8; 24 h, 4 °C), washed (3 × with 50 mM MSB buffer pH 6.8, 4 °C), and dehydrated using an ethanol series (6 × ethanol 30%–100%, 4 °C, 30 min/step). The LR-White resin (London Resin Co., Basingstoke, UK) was used for embedding the tissues. Gelatin capsules filled with resin and the sample were allowed to polymerize in an oxygen-free atmosphere (2 h RT + 36 h 50 °C). Specimens were sectioned into 1 µm-thick sections with a glass knife using the ultramicrotome Ultracut E (Leica-Reichert-Jung, Nussloch, Germany) and mounted onto glass slides. Tissue sections were fixed on slides over a hot plate (70 °C). Staining was carried out with 0.05% toluidine blue for 3–4 min.

## Results

### Generation of early flowering transgenic lines for faster evaluation of gene containment in poplar

Transformation experiments were carried out with two different *Agrobacterium* strains simultaneously: one strain containing the gene construct HSP::*AtFT* (early flowering construct) and the other PsEND1::*barnase–barstar* (gene containment). This strategy was designed to achieve the generation of transgenic lines containing two different T-DNAs after a single genetic transformation. Transgenic lines containing one (single lines: 83) or both constructs (double lines: nine) were obtained (Table S2 available as Supplementary Data). This approach allowed the generation of double transgenic lines. However, most regenerants obtained contained only one T-DNA. Molecular analyses confirmed the presence of gene constructs and the number of T-DNA copies present in the different transgenic lines (Fig. S1 available as Supplementary Data). Southern blot analyses of double transgenic lines showed that two single copy transgenic lines were obtained (transgenic lines N430-4, and N441-7). Other double transgenic lines contained two T-DNA copies (transgenic lines N430-11, N435-15, N435-33, and N441-15) (Fig. S2 available as Supplementary Data).

### Plant growth, flower, and pollen development

**Fig. 1:**
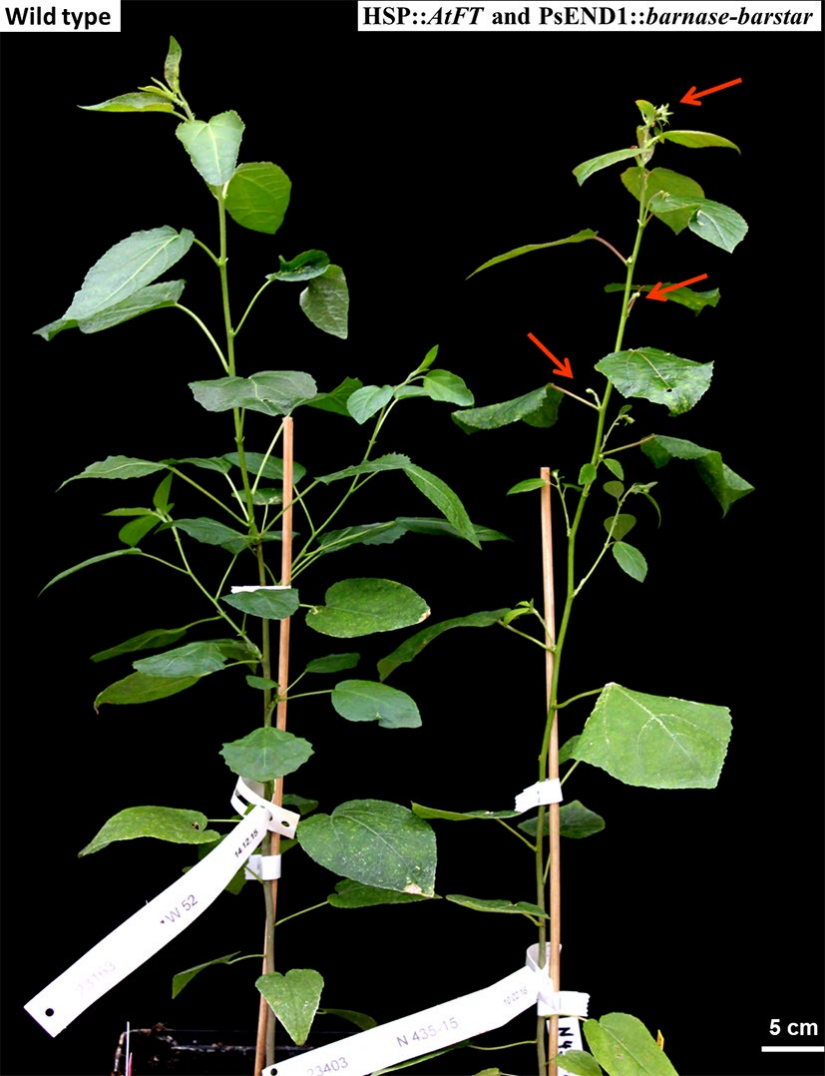
Development of wild type (left) and early flowering transgenic poplar (HSP::*AtFT* + PsEND1::*barnase–barstar*) (right) under growth chamber conditions. Arrows show flowers in transgenic poplar

Nine double transgenic lines were grown under greenhouse conditions for 3–5 months. Plants showed a phenotype without visible effects on vegetative development similar to that of wild-type plants (Fig. 1). From each transgenic line, 5-month-old plants were transferred from the greenhouse to the growth chamber for early flowering induction. After heat treatment, flowers were obtained both in the control (HSP::*AtFT*) and in three double transgenic lines (HSP::*AtFT* and PsEND1::*barnase–barstar*) (Fig. 1).

**Fig. 2:**
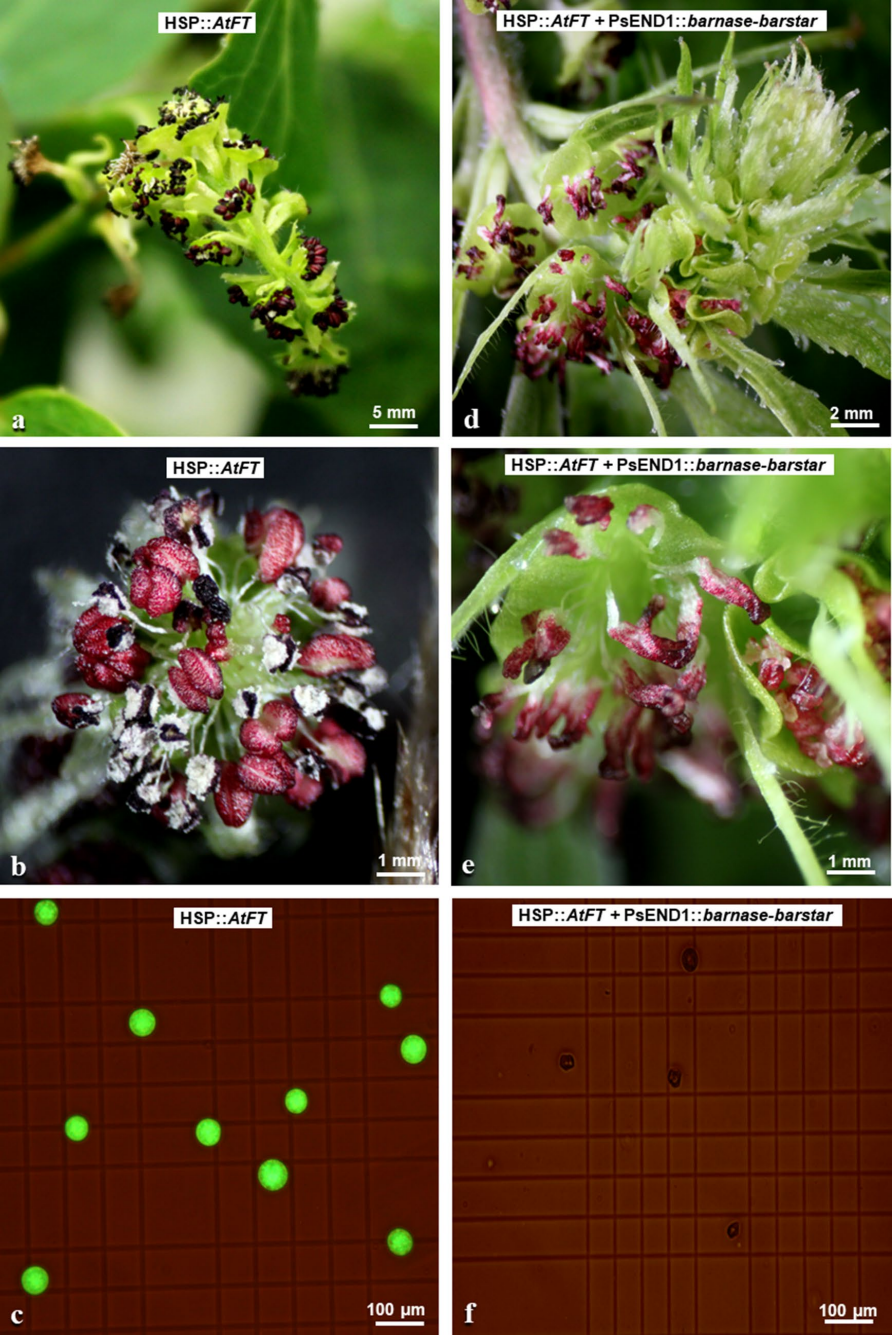
Catkins, anthers, and pollen grains from single and double transgenic lines. Single transgenic lines (HSP::*AtFT*): **a** catkin, **b** flower with anther and pollen grains, and **c** viable pollen grains. Double transgenic lines (HSP::*AtFT* and PsEND1::*barnase–barstar*): **d** catkin, **e** flower with abnormal anthers, and **f** non-viable pollen grains. Microspore viability was estimated by staining with fluorescein diacetate 0.01% (Wildholm 1972). The fluorescence was observed under an optical fluorescence microscope using a mix of white and blue light (490 nm)

After the heat-shock phase, a cold-treatment phase was carried out for the promotion of pollen development in flowers (Hoenicka et al. 2016). The control lines (HSP::*AtFT* poplar) developed flowers with pollen grains (Fig. 2). In contrast, most flowers from double transgenic lines (HSP::*AtFT* and PsEND1::*barnase–barstar* poplar) showed disturbed development and contained no pollen grains (Fig. 2c). However, a few abnormal pollen grains were obtained from anthers of double transgenic lines (Fig. 2c, Tab. 1). Determination of pollen number and viability showed differences between the double transgenic lines (N430-11, N435-15, N441-21) (Table 1); N435-15 anthers had no viable pollen grains, whereas in other transgenic lines, 40% of anthers contained a fraction (2–4%) of the viable pollen grains present in control line anthers (Table 1).

**Table 1:** Presence or absence of pollen grains in anthers and viable pollen grains in single (HSP::*AtFT, flowering* control) and double transgenic lines (HSP::*AtFT* + PsEND1::*barnase–barstar*)

Transgenic line	Gene constructs	Number of anthers with pollen grains (n:20)	Number of pollen grains/anther^a^	Number of viable pollen grains/anther^a^	Proportion of viable pollen grains/anther (%)
T193-2	HSP::*AtFT*	20^b^	274 (21.8)^c^	226 (18.9)^c^	82.5
N430-11	HSP::*AtFT*	8	35 (12.1)	9 (3.7)	25.7
	PsEND1::*barn./bars*				
N435-15	HSP::*AtFT*	1	2 (1.4)	0	0
	PsEND1::*barn./bars*				
N441-21	HSP::*AtFT*	8	18 (5.0)	4 (2.8)	22.2
	PsEND1::*barn./bars*				

Pollen number and viability was measured in 20 anthers/transgenic line

^a^Mean values and standard deviations (SD) are shown

^b^Mean values are significantly higher in single compared to double transgenic lines according to Chi-square test

^c^Two-tailed *t* tests (*p* < 00.001)

**Fig. 3:**
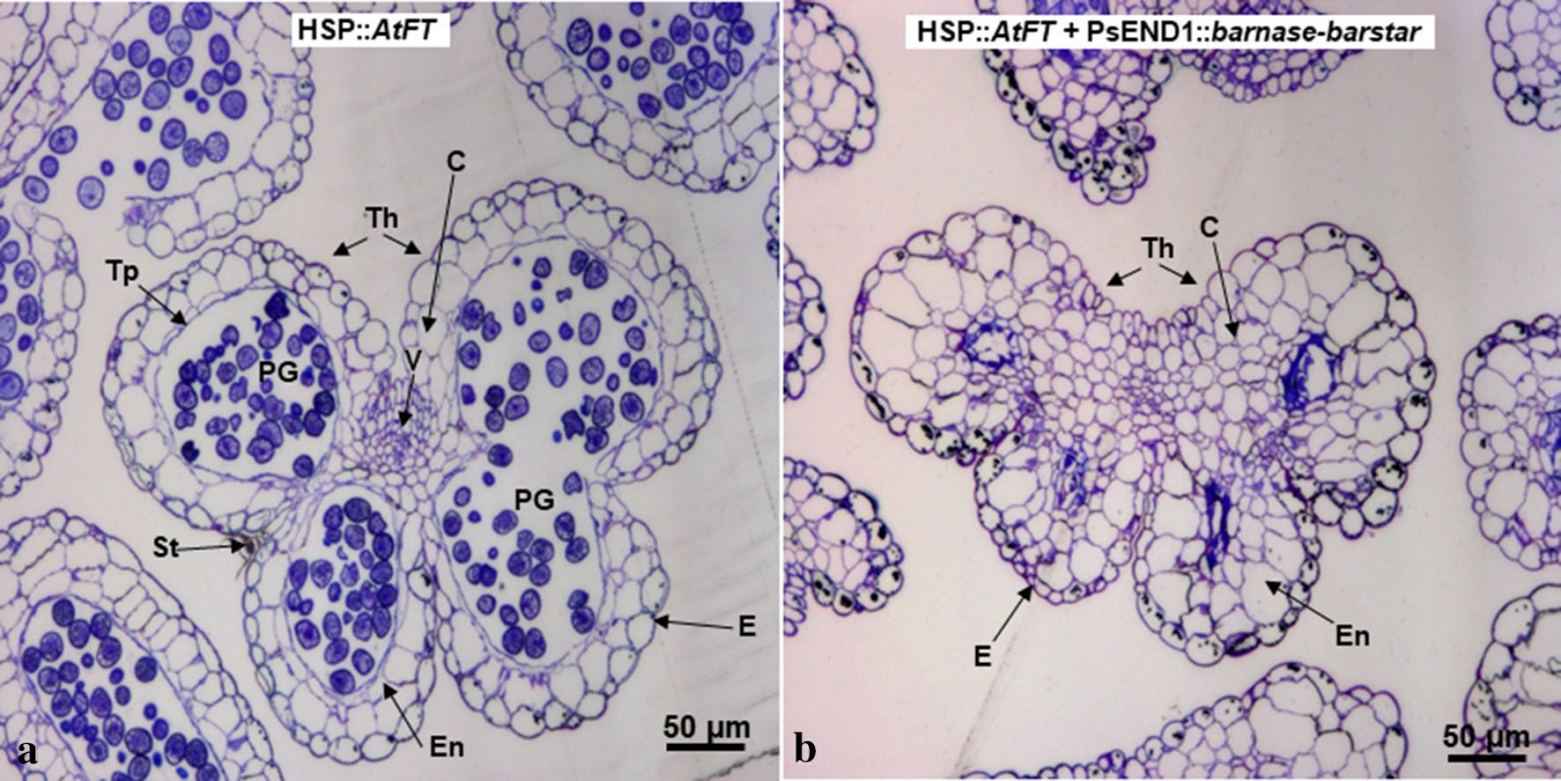
Microscopic study of anthers and pollen grains from single (HSP::*AtFT*) and double transgenic lines (HSP::*AtFT* and PsEND1::*barnase–barstar*). Single transgenic lines **a** showed normal anther and pollen development (stage 14). Double transgenic lines **b** showed strongly disturbed another development, and most anthers lacked viable pollen grains. Anthers were fixed and embedded in LR-White plastic resin and sliced into 1 µm transverse sections. The anther sections were stained with toluidine blue. *C* connective, *E* epidermis, *En* endothecium, *St* stomium, *PG* pollen grains, *V* vascular region, *Th* theca, *Tp* tapetum

Morphological differences between the control plants and the double transgenic lines were assessed based on observations under the microscope. Results showed that control plants (just carrying HSP::*AtFT*) presented an undisturbed anther and pollen development. In these plants, anthers reached the developmental stage 14 (Fig. 3a), according to the scale proposed for *A. thaliana* (Sanders et al. 1999). Mature anthers from double transgenic lines showed an abnormal morphology and no pollen grains were obtained (Fig. 3b).

### Detection of *barnase* gene activity in flowers

**Fig. 4:**
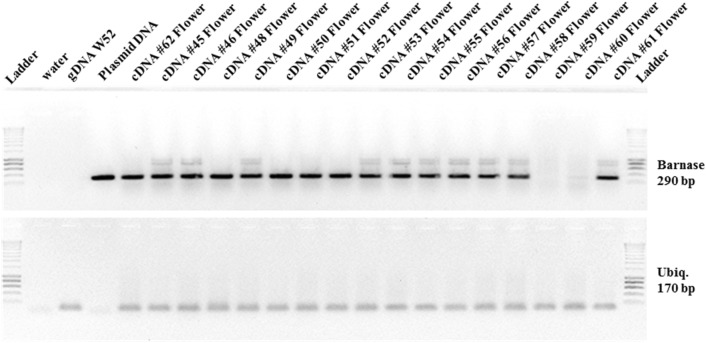
Gene expression analysis of double transgenic lines (HSP::*AtFT* and PsEND1::*barnase–barstar*) by RT-PCR. Expression of the *barnase* gene was confirmed in flowers of double transgenic lines N441-15 and N441-21. *UBQ7* was used as a reference gene

RT-PCRs performed with flowers of double transgenic lines N441-15 and N441-21 (HSP::*AtFT* and PsEND1::*barnase–barstar*) confirmed activity of the *barnase* gene using *UBQ7* as a reference gene. *Barnase* gene activity was confirmed in 15 of 17 studied flowers (Fig. 4).

## Discussion

The strict regulatory requirements for field testing and commercial use of GE trees around the world, gene flow problems with non-native species, and ecological and economic impacts from gene dispersal have led us to search for alternatives towards biosafe release of genetically modified and non-native trees to the environment. Here, we report the production of genetically modified poplars (*P. tremula* L. and *P. tremula* × *P. tremuloides*) lines containing two gene constructs: HSP::*AtFT* for early flowering induction and PsEND1::*barnase–barstar* for genetic containment. The HSP::*AtFT* construct combines a reliable flower induction system with normal vegetative growth (Hoenicka et al. 2016). The mitigation of sexual gene flow provided by the PsEND1::*barnase–barstar* construct in *P. tremula* could enable production of fertile reproductive propagules, and prevent, or reduce, the flow of genes via sexual reproduction, thus offering much needed safer release alternatives.

A variety of recently reported biotechnological strategies are based on ablation of reproductive cells or structures and inactivation or suppression of genes with essential roles in reproduction (Fritsche et al. 2018). Different gene promoters fused to containment genes like *barnase* and *stilbene synthase* were tested previously for genetic containment in poplars (Meilan et al. 2001; Skinner et al. 2003; Hoenicka et al. 2006; Fritsche et al. 2018). Elorriaga et al. (2018) tested the effect of the tobacco TA29 tapetum-specific promoter driving the *barnase gene* on growth rate and male sterility in a field trial of a *P. tremula* × *P. tremuloides* transgenic hybrid poplar. Their results showed strong and consistent but possibly not absolute, male sterility.

This is the first report on the use of the PsEND1::*barnase–barstar* construct in woody plants species. The combination of heat-shock and chilling treatments allows the formation of male fertile flowers in HSP::*AtFT* transgenic poplars, independently of the season (Hoenicka et al. 2016). This system is efficient even in very young plants (4–6 months). That is a very short period of time considering the long poplar vegetative phase (7–12 years).

Transgenic HSP::*AtFT*/PsEND1::*barnase–barstar* plants presented normal vegetative growth, compared with the WT plants grown in vitro and in the greenhouse. Klocko et al. (2016) reported engineered male sterility in pine, poplar, and eucalyptus trees grown under field conditions by expression of the *barnase* gene in the anther tapetal cells. The authors stated that the use of the *barnase* gene can reduce the rates of genetic transformation and vegetative growth. They showed that the use of an RNAi approach to target the poplar homolog of *LFY* resulted in a decrease in inflorescence (catkin) size and loss of functional sexual organs in field-grown trees. However, the transgenic trees reported retained normal vegetative development. In our combined approach, the *barnase* gene was fused to the *barstar* gene, an inhibitor of the Barnase activity, with a non-codifying sequence between both genes. Barnase is a very active ribonuclease and the construct inclusion of the *barstar* gene will prevent ectopic expression of *barnase* and undesirable effects in the regeneration and development of the transgenic plants generated. *Barnase* gene expression was checked in the HSP::*AtFT*/PsEND1::*barnase–barstar* transgenic flowers by RT-PCR analyses, corroborating the utility of this transgene combination for the faster evaluation of gene containment.

In this study, anther development of transgenic plants was abnormal. The construct PsEND1::*barnase–barstar* produced cell ablation in the anther tissues at early developmental stages and consequently male sterility, as reported by Roque et al. (2007, 2019) in two transgenic species of Solanaceae (*Nicotiana tabacum* and *Solanum lycopersicon*), two species of Brassicaceae (*Arabidopsis thaliana* and *Brassica napus*) and two ornamental plants (*Kalanchoe blossfeldiana* and *Pelargonium zonale*). Strategies that use reproductive cells or anther tissues ablation, or the inactivation or suppression of genes essential to the normal reproductive processes have been widely used in woody species.

A cell ablation strategy using the *Bacillus amyloliquefaciens barnase* gene has resulted in male sterility in both softwood and hardwood trees; gene expression was driven by reproductive tissue-specific promoters (Fritsche et al. 2018). Genetic sterility in angiosperm trees was shown via RNAi silencing based on constructs targeting the *LFY* and *AG* flowering genes (Klocko et al. 2018). The *AG* and *LFY* genes appear to be very promising targets for bisexual sterility without an important impact on vegetative development. However, their impact and performance in male clones is unclear, due to the lower rate of RNAi suppression. Targeting both genes using the CRISPR-Cas9 gene-editing technology is expected to be feasible, establishing whether gene knockdown would, indeed, be a universal containment technology in poplar (Klocko et al. 2018).

Early flowering transgenic lines are an efficient and reliable tool for gene containment research in poplar. Our results showed that the PsEND1 promoter has a high potential to prevent undesirable vertical gene flow in this woody plant species. The increasing availability of genome and transcriptome resources for forest trees generating knowledge of reproductive processes and candidate genes for modification, combined with the precision of CRISPR-Cas9 gene-editing-based targeted mutagenesis, suggest that new and more powerful genetic innovations will be achieved in the field of engineered sterility in the near future by offering precise and predictable modifications combined with phenotypic stability.

## Electronic supplementary material

Below is the link to the electronic supplementary material.

Supplementary file1 (DOCX 432 kb)

## Abbreviations

HSP: Heat-shock promoter
PsEND1: *ENDOTHECIUM 1* Gene from *Pisum sativum*
*AtFT*: Flowering-time gene from *Arabidopsis thaliana*
T-DNA: Transfer DNA
GE: Gene editing
