# Supplementary material for: Efficient evaluation of a gene containment system for poplar through early flowering induction
Source: Plant Cell Rep. 2020 Feb 12;39(5):577–87. doi: 10.1007/s00299-020-02515-1 (PMC7165154; doi:10.1007/s00299-020-02515-1)
Supplement: Supplementary file 1 — Supplementary file1 (DOCX 432 kb) [file 299_2020_2515_MOESM1_ESM.docx]

**Efficient evaluation of a gene containment system for poplar through early flowering induction**

M. Valentina Briones^1^, Hans Hoenicka^2^, Luis A. Cañas, José Pío Beltrán, Dieter Hanelt, Sandra Sharry, Matthias Fladung

^1^ Facultad de Ciencias Agrarias y Forestales, Universidad Nacional de La Plata, Argentina; Consejo Nacional de Investigaciones Científicas y Técnicas (CONICET).

2 Thünen Institute of Forest Genetics, D-22927 Grosshansdorf, Germany

Corresponding authors (brionesforestal@gmail.com, hans.hoenicka@thuenen.de)

**SUPPLEMENTARY DATA**

**Table S1** List of primers used in this study.

**Table S2** Genetic transformations. Single and double transgenic lines.

**Figure S1** PCRs analyses of double transgenic lines with specific primers.

**Figure S2** Southern blot analysis with T-DNA (PsEND1::*barnase-barstar*) and END1 probes.

**Table S1** Sequence of primers used in this study (5’- 3’).

| Primer | Forward primer | Reverse primer |
| --- | --- | --- |
| *FT* | AAG TCC TAG CAA CCC TCA CCT C | TGT TTG CCT GCC AAG CTG TC |
| HSP | GAC CAA TCC TAA CCA ATG TCT G | ATA CGA TGC CTT CAC TTC TTT CTG |
| *barnase* | TAT CAA CAC GTT TGA CGG GGT | TGT AAA GGT CTG ATA ATG GTC |
| END1 | TGC GCT AAG GCA TTC AGA GT | CGG ATG CGC AAA GAA ATG CT |
| *UBQ7* | TGA GGC TTA GGG GAG GAA CT | CAA CCC GTT CCT TGA TCC TA |

**Table S2** Results of genetic transformations of poplar with the early flowering (HSP::*AtFT*) and the genetic containment (PsEND1::*barnase-barstar*) gene constructs. Single and double transgenic lines were obtained.

| Transgenic line | Poplar clone | *Regenerants FT* (+) | *Regenerants barnase* (+) | Regenerants  *FT (+)*, *barnase (+)* | Total regenerants |
| --- | --- | --- | --- | --- | --- |
| N426 | T89 | 2 | 0 | 0 | 3 |
| N427 | T89 | 3 | 0 | 0 | 3 |
| N430 | W52 | 20 | 4 | 3 | 23 |
| N431 | W52 | 4 | 0 | 0 | 8 |
| N435 | W52 | 27 | 2 | 2 | 34 |
| N441 | W52 | 16 | 5 | 4 | 18 |
| N442 | W52 | 6 | 0 | 0 | 6 |


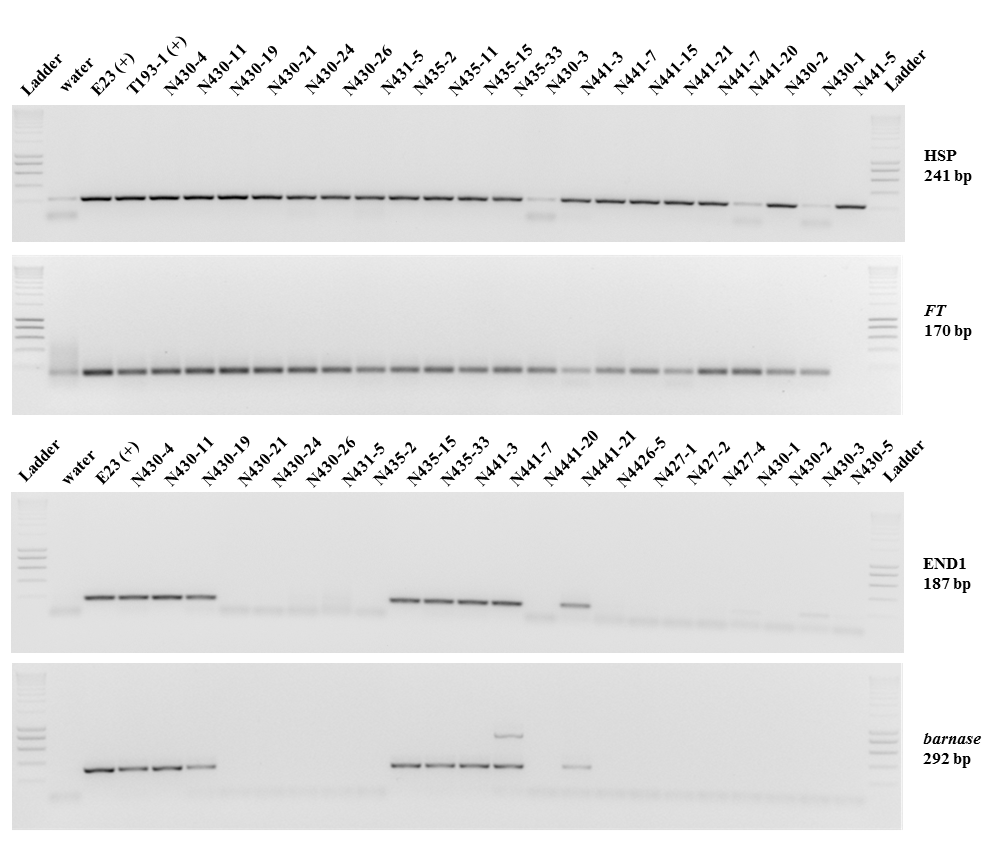


**Fig. S1** PCR analyses of double transgenic lines with specific primers.


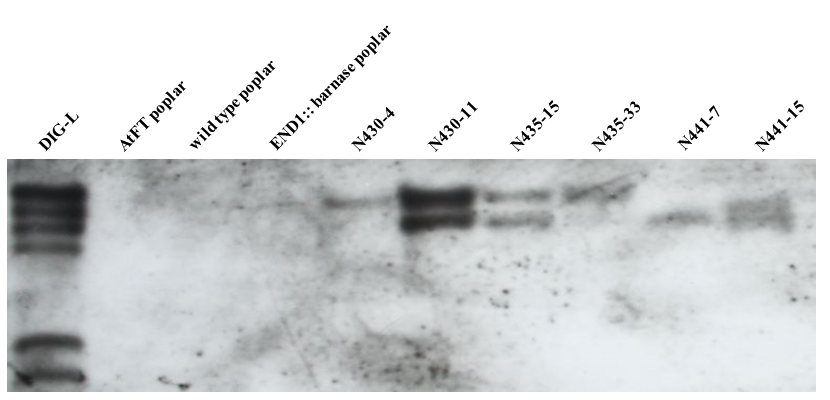


**Fig. S2** Southern blot analysis of double transgenic lines. The DNA was digested with Sac I enzyme and hybridizations were carried out with END1 probe.
